# Correction: Rotational symmetry dication assembling ferroelectric, thermochromic and circularly polarized luminescent multifunctional manganese(ii) bromide hybrid

**DOI:** 10.1039/d6sc90043g

**Published:** 2026-02-23

**Authors:** Jiayi Yuan, Yu Xu, Lu Zhai, Shan-Shan Hei, Jianyi Huang, Weihua Ning, Hong-Ling Cai, Xiao-Ming Ren

**Affiliations:** a State Key Laboratory of Materials-Oriented Chemical Engineering, College of Chemistry and Molecular Engineering, Nanjing Tech University Nanjing 211816 P. R. China zhailu@njtech.edu.cn xmren@njtech.edu.cn; b Institute of Functional Nano & Soft Materials (FUNSOM), Joint International Research Laboratory of Carbon-Based Functional Materials and Devices, Soochow University Suzhou 215123 P. R. China whning@suda.edu.cn; c National Laboratory of Solid-State Microstructures, Collaborative Innovation Center of Advanced Microstructures, School of Physics, Nanjing University Nanjing 210093 P. R. China hlcai@nju.edu.cn

## Abstract

Correction for “Rotational symmetry dication assembling ferroelectric, thermochromic and circularly polarized luminescent multifunctional manganese(ii) bromide hybrid” by Jiayi Yuan *et al.*, *Chem. Sci.*, 2026, https://doi.org/10.1039/d5sc08499g.

The authors regret that incorrect details were given for ref. 45 in the original article. The corrected version of ref. 45 can be found below as ref. 1.

The Royal Society of Chemistry apologises for these errors and any consequent inconvenience to authors and readers.
